# Identification of a two-component Class IIb bacteriocin in *Streptococcus pyogenes* by recombinase-based *in vivo* expression technology

**DOI:** 10.1038/srep36233

**Published:** 2016-11-03

**Authors:** Brent D. Armstrong, Christine A. Herfst, Nicholas C. Tonial, Adrienne T. Wakabayashi, Joseph J. Zeppa, John K. McCormick

**Affiliations:** 1 Department of Microbiology and Immunology, Schulich School of Medicine and Dentistry, Western University, London, ON, Canada; 2 Lawson Health Research Institute, London, Ontario, Canada

## Abstract

*Streptococcus pyogenes* is a globally prominent bacterial pathogen that exhibits strict tropism for the human host, yet bacterial factors responsible for the ability of *S. pyogenes* to compete within this limited biological niche are not well understood. Using an engineered recombinase-based *in vivo* expression technology (RIVET) system, we identified an *in vivo*-induced promoter region upstream of a predicted Class IIb bacteriocin system in the M18 serotype *S. pyogenes* strain MGAS8232. This promoter element was not active under *in vitro* laboratory conditions, but was highly induced within the mouse nasopharynx. Recombinant expression of the predicted mature *S. pyogenes* bacteriocin peptides (designated SpbM and SpbN) revealed that both peptides were required for antimicrobial activity. Using a gain of function experiment in *Lactococcus lactis*, we further demonstrated *S. pyogenes* immunity function is encoded downstream of *spbN*. These data highlight the importance of bacterial gene regulation within appropriate environments to help understand mechanisms of niche adaptation by bacterial pathogens.

*Streptococcus pyogenes* (Group A *Streptococcus*) is a human-adapted bacterial pathogen responsible for non-invasive infections such as pharyngitis and impetigo, severe invasive diseases including necrotizing fasciitis and toxic shock syndrome, and post-infection autoimmune disorders such as rheumatic heart disease^1^. Global morbidity and mortality due to *S. pyogenes* is substantial, previously estimated at over 600 million throat infections, at least 18 million invasive infections, and more than 500,000 deaths each year^2^,^3^. Despite this massive burden of disease, the biological niche of *S. pyogenes* represents a state of asymptomatic colonization on the skin and in the nasopharynx^4^. Although *S. pyogenes* has evolved multiple mechanisms to subvert host immune responses and cause disease^5^,^6^,^7^, it remains unclear how this bacterial pathogen successfully competes with the numerous members of the endogenous microbiota within the nasopharyngeal microenvironment.

In order to gain a more complete understanding of the molecular basis of *S. pyogenes* niche adaptation, we engineered a recombinase-based *in vivo* expression technology (RIVET) system^8^ using the Cre-*loxP* system^9^ to identify genes that are specifically induced within a model *in vivo* environment. Variations of this ‘promoter trap’ strategy have now been successfully applied to a variety of important bacteria including *Vibrio cholera*^8^,^10^, *Mycobacterium tuberculosis*^11^, *Bordetella pertussis*^12^, *Staphylococcus aureus*^13^, *Lactobacillus plantarum*^14^, and *Enterococcus faecalis*^15^. In this work, we integrated an engineered *loxP*-flanked tetracycline resistance cassette (*tetR*) into a neutral site within the *S. pyogenes* chromosome. We demonstrate robust stability of the cassette in the absence of the Cre recombinase, and complete and specific resolution of the cassette under artificial Cre expression. We subsequently screened a random chromosomal library of potential promoter fragments by growth in standard laboratory medium, and an *in vivo* model of acute nasopharyngeal infection. Using this system, we identified a promoter that was located upstream of a potential Class IIb bacteriocin system annotated as the bacteriocin-like peptide (*blp*) operon that was very weakly expressed *in vitro*, but highly induced *in vivo*. Here we designate these genes as the *S. pyogenes* bacteriocin (*spb*) locus, and show that this system encodes active antimicrobial peptides and immunity function, providing a potential novel mechanism by how *S. pyogenes* may compete with other members of the nasopharyngeal microbiota.

## Results

### Construction of the *loxP-tetR-loxP* RIVET cassette in *S. pyogenes* MGAS8232

**Figure 1:**
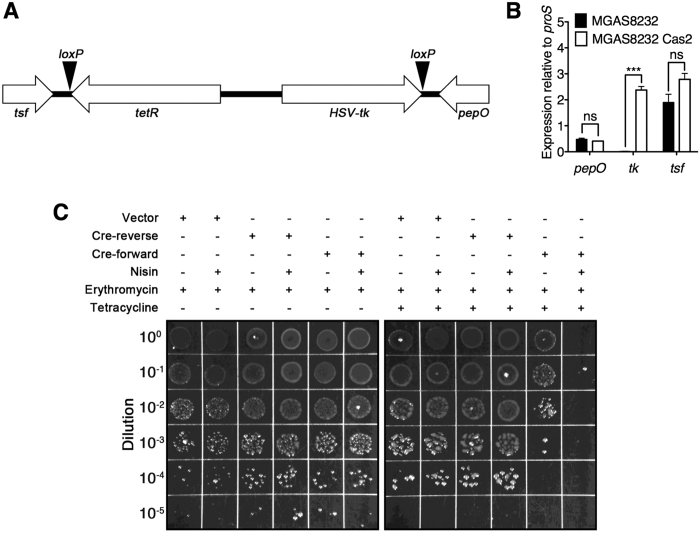
Overview and functionality of the RIVET system in *S. pyogenes*. (**A**) Scale schematic of the 2976-bp RIVET cassette containing an antibiotic resistance cassette (*tetR*) and herpes virus thymidine kinase (*HSV-tk*), flanked by two *loxP* sequences and inserted between the *tsf* and *pepO* gene in *S. pyogenes*. (**B**) qRT-PCR analysis of the RIVET cassette integration site comparing wild-type *S. pyogenes* MGAS8232 with MGAS8232 Cas2. Data represents the mean ± SEM of three biological replicates and statistical significance is displayed as ***p < 0.001 by Student’s *t*-test. (**C**) *S. pyogenes* Cas2 containing the RIVET cassette was transformed with empty vector (pMSP3535), or pMSP3535 containing *cre* in the reverse (pMSP3535::*cre*-reverse) or forward (pMSP3535::*cre*-forward) orientations, both under the control of the nisin-inducible promoter^18^. Strains were grown with or without the addition of nisin (100 ng/ml) as indicated and plated on THY agar containing erythromycin or erythromycin and tetracycline. Dilutions of bacteria prior to plating are shown on the left.

In order to engineer a RIVET system in *S. pyogenes*, we integrated a *loxP*-flanked antibiotic resistance cassette into the chromosome of *S. pyogenes* MGAS8232. We first selected a 165-bp intergenic region suitable for insertion of foreign DNA sequences. This intergenic region was located between two previously predicted Rho-independent transcriptional terminators^16^, each located downstream of the divergently transcribed endopeptidase O (*pepO)* and elongation factor Ts (*tsf*) genes. This region is highly conserved among other sequenced genomes of *S. pyogenes* and may represent a suitable location for *in trans* chromosomal complement experiments. We then integrated an engineered 2,976-bp gene cassette into the *tsf/pepO* intergenic region using the pG^+^host5 temperature sensitive integration vector^17^. This gene cassette was flanked by *loxP* recognition sites for the Cre-recombinase, and contained both a tetracycline resistance (*tetR*) gene, as well as a *S. pyogenes* codon optimized thymidine kinase from Herpes simplex virus (*HSV-tk*) (Fig. 1A). The *HSV-tk* gene was engineered initially for use as a counter selection system in conjunction with ganciclovir for the second round of the RIVET procedure; unfortunately, this gene was only intermittently functional in *S. pyogenes* and although *HSV-tk* was included in the cassette, we instead relied on colony patching for tetracycline sensitivity during the second round of screening (described below). The correct integration of the *loxP-tetR-loxP* cassette was verified by PCR and DNA sequencing, and this clone was designated *S. pyogenes* MGAS8232 Cas2. Furthermore, we also confirmed similar transcription levels of *pepO* and *tsf* by qPCR in wild-type MGAS8232 and MGAS8232 Cas2 (Fig. 1B).

Next, to evaluate the functionality of the *loxP-tetR-loxP* cassette, we transformed MGAS8232 Cas2 with the erythromycin resistant pMSP3535 vector containing the nisin inducible promoter^18^, and pMSP3535 containing *cre* in the forward direction (pMSP3535::*cre*-forward). As an additional control, we also generated a construct containing *cre* in the reverse orientation to the nisin promoter (pMSP3535::*cre*-reverse). Transformants were grown in liquid THY, and separately in THY containing 100 ng/ml nisin, and plated on erythromycin, or erythromycin and tetracycline containing media to assess cassette resolution under Cre induction. The addition of nisin did not result in the loss of tetracycline resistance for *S. pyogenes* Cas2 containing either the pMSP3535 vector, or the pMSP3535::*cre*-reverse plasmid, whereas colonies were not recovered from cells containing pMSP3535::*cre*-forward under tetracycline selection (Fig. 1C). Some tetracycline resistance was also lost in the absence of nisin in cells containing pMSP3535::*cre*-forward; this can be explained by apparent leakiness of the nisin promoter in the absence of induction. Furthermore, sequencing across the resolved cassette region revealed a precise excision of the cassette leaving only a single *loxP* site. These data indicate that a functional *loxP-tetR-loxP* RIVET cassette was engineered into the chromosome of *S. pyogenes* MGAS8232, and that this cassette is stable in the absence of Cre expression under *in vitro* growth conditions.

### Generation of a *S. pyogenes* MGAS8232 promoter RIVET library

To generate a plasmid for use in the promoter library, we cloned the *cre* gene into the low-copy *E. coli*/Gram-positive bacterial shuttle vector pTRKL2^19^. Transformation of pTRKL2::*cre* into *S. pyogenes* Cas2 did not result in detectable cassette resolution based on the tetracycline resistance phenotype. Next, genomic DNA from wild-type MGAS8232 that was partially digested with Sau3AI was ligated into the promoterless pTRKL2::*cre*, and the library was transformed into *E. coli* XL1-Blue. PCR of random clones from the library was used to verify that a variety of fragments were inserted and most clones contained inserts between 40-bp to ~1000-bp. Clones were subsequently pooled from plates, and plasmids were isolated and transformed as a mixture into *S. pyogenes* Cas2. Cells were subsequently grown in liquid for 48 h in the presence of erythromycin for plasmid maintenance, and tetracycline to remove any cells that had resolved the cassette through the induction of Cre via an *in vitro-*active promoter region. These cells were then pooled, enumerated, and frozen until used in the *in vivo* nasopharyngeal infection model.

### Identification of *S. pyogenes* MGAS8232 promoter elements induced *in vivo* using an acute nasopharyngeal infection model

Approximately 1 × 10^8^ cells from the promoter library in *S. pyogenes* Cas2 were used to inoculate the nasal passages of mice as described in *Methods*. After 48 h, mice were euthanized and the nasal passages were harvested as described^20^, and bacteria were plated onto media containing erythromycin. Individual clones were subsequently patched onto media containing tetracycline to identify tetracycline-sensitive clones with resolved cassettes, and therefore potentially containing *in vivo*-induced promoters. Plasmids were extracted from a total of 177 tetracycline sensitive clones, inserts were sequenced, and BLASTn was used to match the putative promoter sequences to the *S. pyogenes* MGAS8232 genome. Strikingly, none of the 177 clones lacked an insert. From this pool, removal of duplicated sequences produced 82 clones containing unique sequences. However, within this pool we also obtained 21 clones that contained multiple inserts, likely due to cloning artifacts, and due to potential transcriptional read-through the analysis of these clones was compromised and they were not included within our dataset. In total, we obtained 61 unique clones with single inserts, and each clone is listed in Table S1. Based on the sequence orientation and location relative to annotated ORFs, we found three types of clones, similar to those described previously^15^. These included ‘typical promoters’ that contained sequences within the 5′-untranslated regions upstream of an ORF, ‘cryptic promoters’ that were found entirely within, and in the same orientation, of an ORF, and ‘antisense promoters’ that were located in the opposite orientation to annotated ORFs. From this pool, we identified 9 sequences consistent with ‘typical’ promoters, located upstream of open reading frames and these are summarized in Table 1. Although we have no additional evidence that the remaining clones represent true *in vivo*-induced promoters, this initial categorization may prove useful for future studies involving anti-sense regulation in *S. pyogenes*.

### *P*_*spbM*_ is an *in vivo*-induced promoter

From the RIVET screen, we selected clone IVI156 for further analysis (Table 1). The plasmid from IVI156 contained a 984-bp sequence upstream and encompassing the 5′ end of SPYM18_RS02350, a potential bacteriocin peptide structural gene homologous to *blpM* from *Streptococcus pneumoniae*^21^. Immediately downstream of SPYM18_RS02350 was an additional ORF (SPYM18_RS02355) with homology to *S. pneumoniae blpN*^21^. As described below, and to avoid confusion between the two systems, we designate these ORFs as the *S. pyogenes* bacteriocin M (*spbM*) and *spbN* genes.

**Figure 2:**
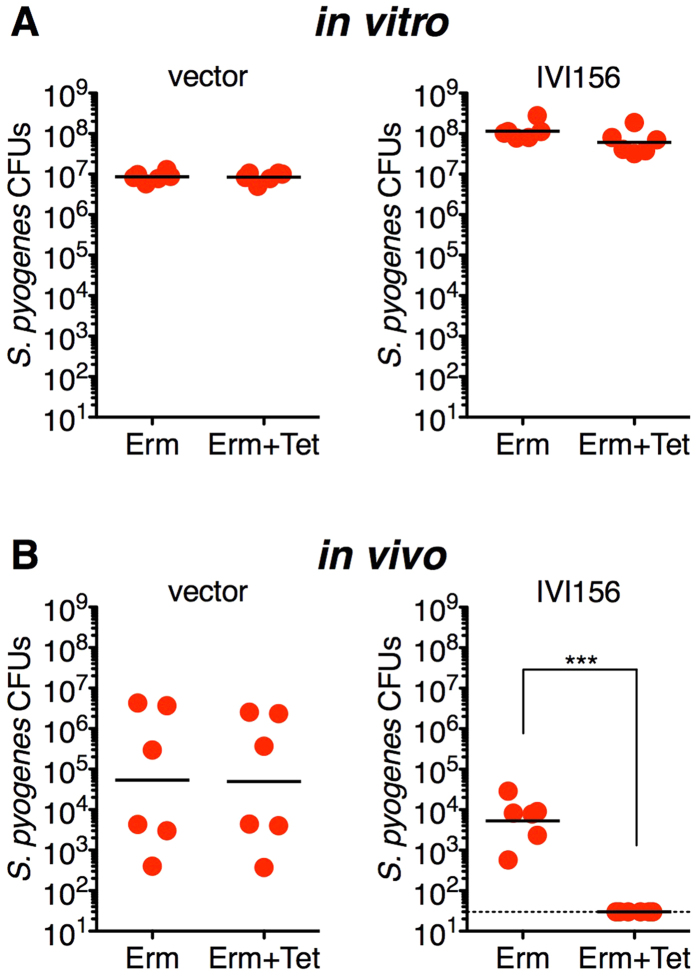
The *S. pyogenes* bacteriocin promoter (P_*spbM*_) is an *in vivo*-induced promoter. *S. pyogenes* Cas2 containing pTRKL2::*cre* (vector control) or *S. pyogenes* clone IVI156 (encoding the P_*spbM*_ promoter) were assessed for cassette resolution by growing cells under *in vitro* or *in vivo* conditions as described in *Methods*. For *in vivo* conditions, mice were infected with 1 × 10^8^ CFUs of *S. pyogenes* intranasally for 48 hours. Mice were then sacrificed and the complete nasal turbinates were harvested for bacterial enumeration. Each dot represents CFUs from an individual experiment, and cells were plated on THY agar containing erythromycin, or erythromycin plus tetracycline, and CFUs determined. The black bar represents the geometric mean and statistical significance is displayed as **p < 0.001 by Student’s *t*-test. The dotted line represents the theoretical limit of detection.

The plasmid from clone IVI156 was designated pTRKL2::P_*spbM*_::*cre*. This plasmid, and the vector control (pTRKL2::*cre*) were retransformed into *S. pyogenes* Cas2 and clones were grown *in vitro* for 24 h in THY broth. Neither the vector control or pTRKL2::P_*spbM*_::*cre* induced detectable excision of the *loxP-tetR-loxP* cassette as determined by comparing CFUs on medium containing erythromycin, or medium containing both erythromycin and tetracycline (Fig. 2A). Next, we conducted the nasopharyngeal infection model using *S. pyogenes* Cas2 containing either the pTRKL2::*cre* vector, or pTRKL2::P_*spbM*_::*cre*, and following 48 h, harvested the complete nasal turbinates and plated cells directly on medium containing erythromycin, or erythromycin and tetracycline. As expected, *S. pyogenes* Cas2 containing the promoterless pTRKL2::*cre* plasmid did not induce excision of the *loxP-tetR-loxP* cassette (Fig. 2B). Although *S. pyogenes* Cas2 containing pTRKL2::P_*SpbM*_::*cre* trended to infect the mice at lower levels compared with Cas2 containing the vector control, the infection with *S. pyogenes* Cas2 containing pTRKL2::P_*SpbM*_::*cre* resulted in a complete loss of tetracycline resistant colonies (Fig. 2B). These data provide important evidence that P_*spbM*_ was not functionally expressed in this system under *in vitro* conditions, but that P_*spbM*_ was induced during the model nasopharyngeal *in vivo* environment.

**Figure 3:**
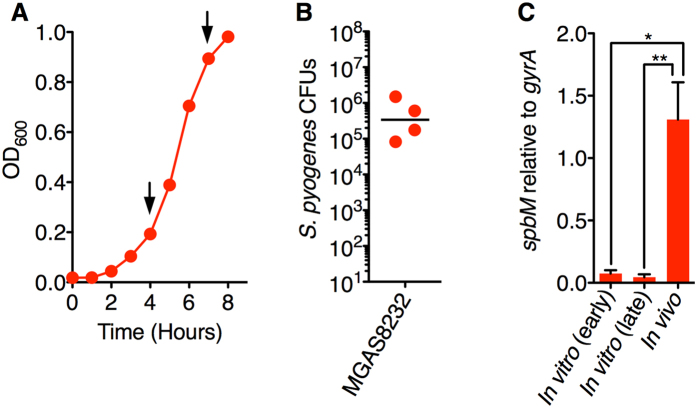
Analysis of *spbM* gene expression *in vitro* and *in vivo*. *S. pyogenes* MGAS8232 was grown (**A**) in THY broth to OD_600_ ~0.2 or ~0.9 (n = 3; early and late *in vitro* conditions, respectively) or (**B**) nasally inoculated into mice and harvested at 48 h (n = 4; *in vivo* conditions) as described in *Methods*. The black bar represents the geometric mean. (**C**) Expression of *spbM* was determined by qRT-PCR and normalized to the *gyrA* housekeeping gene. The graph shows the mean ± SEM and statistical significance is displayed as *p < 0.05 by ANOVA with Tukey’s Multiple Comparison Test.

To confirm that P_*spbM*_ was in fact induced *in vivo*, we conducted qRT-PCR experiments of *spbM* transcripts from wild-type *S. pyogenes* MGAS8232 grown under *in vitro* (Fig. 3A) or *in vivo* (Fig. 3B) conditions. Consistent with the data using MGAS8232 Cas2 containing pTRKL2::P_*spbM*_::*cre*, *spbM* transcripts were very weakly expressed *in vitro* relative to the *gyrA* housekeeping gene, yet *spbM* transcripts were significantly increased at 48 h from the *in vivo* nasopharyngeal infection model (Fig. 3C). These data provide direct evidence that *spbM* from wild-type MGAS8232 was specifically induced in the *in vivo* nasopharyngeal environment.

### Bioinformatic analysis of the *S. pyogenes spb* operon

**Figure 4:**
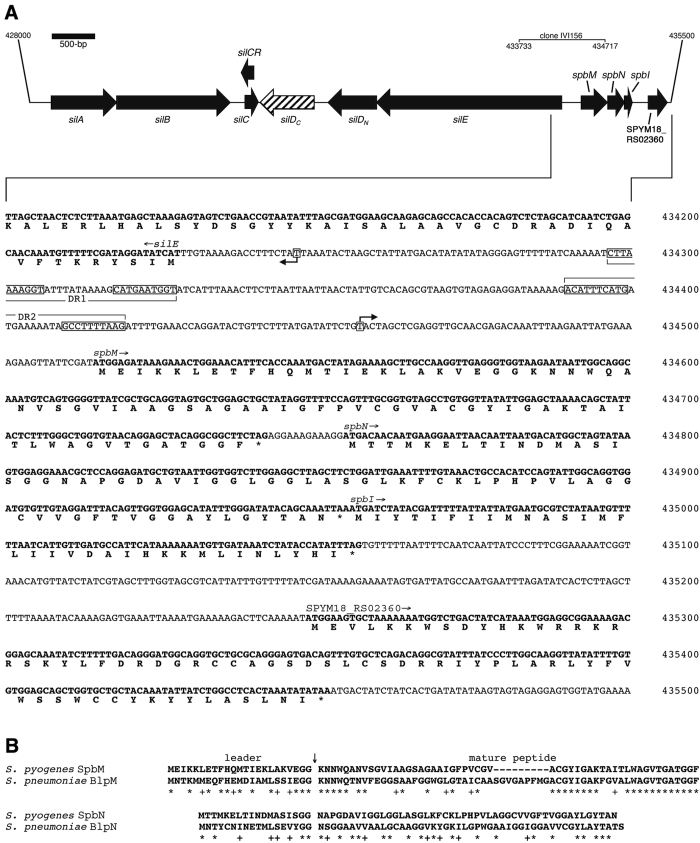
The *sil-spb* loci in *S. pyogenes* MGAS8232. (**A**) Open reading frame map and DNA sequence of the *sil* and *spb* operons. The *silD* gene in MGAS8232 contains a premature stop codon and may potentially encode two separate ORFs labeled *silD*_*N*_ and *silD*_*C*_ (the latter denoted with hashed lines). The nucleotide and translation products for the *spb* locus are given below for the indicated region. The location of the DNA region contained within clone IVI156 is indicated. Promoter regions are drawn as black arrows and direct repeats (DR1 and DR2) are annotated as previously determined in *S. pyogenes* JS95^42^. Nucleotide numbers are according to the genome sequence of *S. pyogenes* MGAS8232 (NC_003485.1). (**B**) Alignment of the Spb peptides from *S. pyogenes* MGAS8232 and Blp peptides from *S. pneumoniae* 6A^21^. Identical (*) and similar (+) residues are indicated. The arrow (↓) indicates the predicted cleavage site of the double-glycine-type leader peptides.

Clone IVI156 contained sequences consistent with a known promoter region driving the expression of *spbM* in *S. pyogenes* JS95^22^, and is adjacent and in opposite orientation to the streptococcal invasion locus (*sil*)^23^ (Fig. 4A). Class IIb bacteriocins exert optimal antibacterial activity when two peptides, encoded in tandem, are present^24^. The *spbM* and downstream *spbN* genes encode for peptides that share hallmarks of the Class IIb bacteriocins, including GXXXG motifs^24^. SpbM and SpbN contain four and five GxxxG motifs, respectively, which allow for helix-helix interactions between the two peptides to create a membrane penetrating structure^24^. The peptide sequence encoded by the *spbM* gene in *S. pyogenes* MGAS8232 showed 50% identity to BlpM from *S. pneumoniae*, while the peptide sequence encoded by *spbN* in *S. pyogenes* showed 35% identity to the *S. pneumoniae* BlpN^21^ (Fig. 4B). In addition, most Class II bacteriocins require a dedicated transport apparatus that recognizes and cleaves a specialized ‘double-glycine-type’ leader peptide^25^. Both *S. pyogenes* SpbM and SpbN, as with the *S. pneumoniae* system, contain sequences consistent with double-glycine-type leader peptides (Fig. 4B). For the bacteria to prevent self-killing by the bacteriocin, the producing organism encodes an immunity peptide that is invariably linked to the structural bacteriocin genes. It is likely, based on typical Class II bacteriocin operon structure^24^, that one of the genes located downstream from *spbN* encodes this function, similar to the *S. pneumoniae* system^21^. Downstream of *spbN* are two potential ORFs each which may encode immunity function to the SpbMN bacteriocin (Fig. 4A). The first ORF is not annotated in the MGAS8232 genome, while the second ORF is designated SPYM18_RS02360.

In addition to the bacteriocin structural and immunity genes, Class IIb bacteriocins also require dedicated transporters, and additionally, often encode proteins involved in self-regulation^21^. Upstream of *spbM* in MGAS8232 was a group of genes that correspond to the streptococcal invasion locus (*sil*)^23^. The *sil* locus is comprised of an ATP-binding cassette (ABC) transporter (*silDE*), a pheromone (*silCR*), a peptide (*silC*), and a two-component system (*silAB*). The *sil* locus has been shown to be important for invasive infections^23^,^26^ and can regulate *spbMN* and its associated immunity gene in *S. pyogenes* JS95^22^. MGAS8232 encodes a full-length *silE* predicted to produce a 717 amino acid ATP-binding cassette transporter. However, in MGAS8232 *silD* (SPYM18_RS02340) appears to be a truncated form of the complete 454 amino acid SilD, which normally works in conjunction with SilE to form an ABC transporter complex. Analysis of the nucleotide sequence revealed that a deleted adenine would predict early termination of MGAS8232 *silD* (designated here as *silD*_*N*_). Although a potential downstream ORF could theoretically encode the C-terminus of this protein (designated here as *silD*_*C*_), there were no obvious translation initiation sequences preceding *silD*_*C*_ and this hypothetical ORF is likely not translated (Fig. 4A).

### The Spb operon encodes a functional Class IIb two-peptide bacteriocin

Although Class II bacteriocins, to our knowledge, have not been characterized from *S. pyogenes*, this organism is well known to be capable of producing lantibiotic-type Class I bacteriocins, including salivaricin A (SalA)^27^, and streptin (StrA)^28^. MGAS8232 does encode salivaricin A^29^, although the operon contains a large deletion in the *salM* and *salT* genes^27^. Nevertheless, we tested wild-type MGAS8232 for bacteriocin production *in vitro* using a standard deferred antagonism assay against a defined set of 9 bacteriocin indicator strains^30^; however, we did not detect any bacteriocin activity from MGAS8232 *in vitro*.

**Figure 5:**
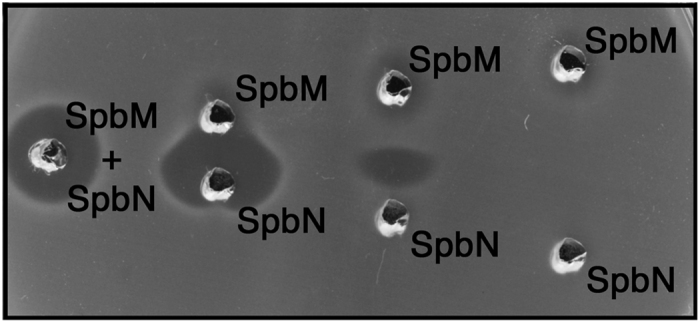
The Spb operon encodes functional two-component bacteriocin peptides. Recombinant SpbM and SpbN peptides (~1 μg per well) were spotted into wells at increasing distances punched into agar inoculated with SpbMN-sensitive *L. lactis* MG1363 and the indicator lawn was allowed to grow over night at 30 °C.

Since transcription of the *spb* system in MGAS8232 was barely detectable *in vitro* (Fig. 3), in order to assess the ability of SpbM and SpbN from MGAS8232 to function as a Class IIb bacteriocin, we produced recombinant mature peptides from *E. coli*. A series of indicator strains were tested by 1% inoculation of molten 1.5% agar medium, followed by punching wells into the solidified agar. SpbM alone demonstrated weak activity against a number of indicators whereas SpbN alone did not display any detectable antimicrobial activity. When SpbM and SpbN were mixed however, clear zones of inhibition were seen against multiple *S. pyogenes* strains (MGAS8232, MGAS5005, SF370), as well as strains of *Streptococcus dysgalactiae*, *Streptococcus uberus*, *Micrococcus leuteus* and *Lactococcus lactis*. To further evaluate the two-component nature of SpbM and SpbN, we placed these recombinant peptides (~1 μg each) at increasing distances within wells on agar plates and evaluated antimicrobial activity using *L. lactis* as an indicator. As demonstrated in Fig. 5, optimal antimicrobial activity required the presence of both SpbM and SpbN indicated by zones of inhibition where the two peptides had diffused together. These data demonstrate that the SpbMN peptides can function as a Class IIb bacteriocin.

### Spb immunity function is encoded downstream of *spbN*

**Figure 6:**
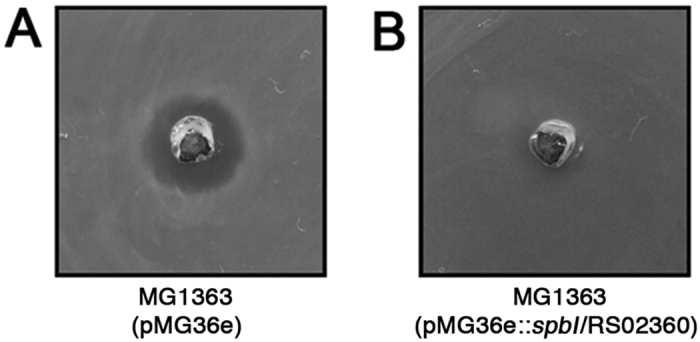
Immunity function to the *S. pyogenes* SpbMN bacteriocin is encoded within the *spb* operon. Recombinant SpbM and SpbN peptides (1 μg each) were added to wells punched into agar inoculated with (**A**) *L. lactis* containing control plasmid pMG36e or (**B**) *L. lactis* containing pMG36e::*spbI/RS02360.*

In order to assess potential immunity function encoded within the *spb* operon, we conducted gain-of-function experiments in the SpbMN sensitive host *L. lactis* MG1363. We first cloned the potential ORF immediately downstream of *spbN* into the Gram-positive expression vector pMG36e, which contains the constitutive lactococcal promoter P32^31^. This plasmid did not induce resistance to SpbMN when transformed into *L. lactis* MG1363 (data not shown). We then generated a construct that contained both this gene, and SPYM18_RS02360, which we designate pMG36e::*spbI/*RS02360. When pMG36e::*spbI/*RS02360 was transformed into *L. lactis* MG1363, these cells became resistant to recombinant SpbMN compared with cells containing the pMG36e vector (Fig. 6). This data indicates that immunity function to SpbMN is encoded downstream of *spbN*.

## Discussion

Humans are the only recognized niche for *S. pyogenes*, and the upper respiratory tract and skin represent the dominant reservoirs for this globally important pathogen^32^. In this work, we adapted the RIVET strategy^8^ to isolate *in vivo*-activated promoters in *S. pyogenes* using a model of upper respiratory tract infection to help understand the genetic mechanisms that contribute to infection and persistence. A key advantage to utilizing RIVET over other genomic technologies is the ability to select transiently expressed genes during the course of the environmental exposure that could be missed using genomic technologies that rely on specific time points. Additionally, RIVET can also theoretically detect bacterial genes induced from minor populations of heterogeneous (non-synchronous) cells. Overall, our analysis generated 61 unique clones with single inserts from *S. pyogenes* MGAS8232 that had apparently resolved the *loxP-tetR-loxP* cassette *in vivo* (Table S1).

From the group of ‘sense’ clones, nine appeared to represent typical promoters, likely driving the expression of a gene(s), including clone IVI156 that would control the expression of the *spb* operon. Bacteriocins from Gram-positive bacteria are small, ribosomally synthesized antimicrobial peptides that typically demonstrate activity against closely related organisms^33^. There are three major classes of bacteriocins including: Class I bacteriocins (<10 kDa), also termed lantibiotics, that undergo post-translational modification to incorporate lanthionine or β-methyllanthionine residues into the active peptide; Class II bacteriocins (<10 kDa) that are typically not post-translationally modified except for leader peptide cleavage and disulfide bond formation; and Class III bacteriocins (>10 kDa). It is widely thought that bacteriocins exist as factors to compete with other bacteria for ‘space’ within a niche^21^,^34^. Since the upper respiratory tract in humans contains a highly complex microbiota^35^, including many species of streptococci^36^, it is not surprising that organisms that exist within this environment produce bacteriocins. It has also been demonstrated that endogenous or exogenous (e.g. probiotic) oral streptococci can compete with *S. pyogenes*, potentially through the use of bacteriocins^37^, although to our knowledge, there is no direct evidence as to how *S. pyogenes* itself can compete within this complex microbiota. However, the homologous BlpM/BlpN system in *S. pneumoniae* strain 6A has been shown to be functionally active and important for intraspecies competition *in vivo*^21^ and it will be of interest in future work to compare these systems for the ability to cross complement in terms of both antimicrobial and immunity function.

Although the *spb* operon has been annotated as ‘Blp-like”, to our knowledge this work is the first to formally demonstrate functional Class IIb bacteriocin peptides in *S. pyogenes*. Following the re-passaging of the *S. pyogenes* Cas2 containing pTRKL2::P_*spbM*_::*cre* through the *in vivo* model produced a striking reduction in tetracycline resistant clones (Fig. 2). In 6 independent mice, we were unable to recover clones with an intact cassette indicating that P_*spbM*_ was uniformly induced *in vivo*, at least in cells that were recoverable from the *in vivo* environment. However, our analysis also indicates that the *sil* locus is not likely operational in MGAS8232 given the apparent truncation of *silD*. Interestingly, and as previously noted, most strains of *S. pyogenes* actually lack the majority of the *sil* locus^38^; however, other than *S. pyogenes* M1 serotypes which contain an 11-bp deletion resulting in a frame-shift mutation within the *spbM* coding sequence [e.g. strains SF370^39^, MGAS5005^40^ and M1_476^41^], most sequenced *S. pyogenes* strains contain complete *spbM* and *spbN* sequences. Furthermore, two direct repeats (DRs) in the *spbM* promoter region have been shown to be important for regulation by the SilA and SilB two-component system^42^, and that the length of an 11-bp spacer between both DRs was critical for function. Verified via sequencing, MGAS8232 contains a 10-bp spacer length in DR2 (Fig. 4A), and when the spacer was shortened to 10-bp in *S. pyogenes* JS95, promoter activity was eliminated. Therefore, although a functional *sil* locus is clearly able to regulate *spbM* in some strains of *S. pyogenes*^22^,^42^, our work suggests that an additional regulatory system that can respond to an *in vivo* signal is able to activate the *spbM* promoter. Given the truncation of *silD* in MGAS8232, it remains unclear as to how the SpbMN peptides would gain access to the extracellular milieu. Given the frequent conservation of the *spb* system in the absence of *sil*, the Spb peptides could be exported through a currently unidentified transporter. Alternatively, given the nasopharyngeal environment where this system is induced, bacterial cell lysis mediated through the immune system, other competing microbes, and/or potentially the induction of lysogenic bacteriophage from the genome of *S. pyogenes*, could each provide a mechanism by which the SpbMN peptides are released. However, immature Class II bacteriocins are only weakly active, while removal of the double-glycine-type leader greatly enhances antimicrobial activity^43^,^44^. Processing of bacteriocin double-glycine-type leaders is thought to be coupled with export via an ATP-binding cassette (ABC) translocator which commonly contains a N-terminal peptidase belonging to the C39 family of peptidases^43^,^45^. Utilizing the MEROPS database^46^, *S. pyogenes* MGAS8232 contains a C39 peptidase motif within SilE, and an additional C39 peptidase motif in an ORF located ~5500 bp upstream of *silA* (locus tag SPYM18_RSO2285). The peptidase domain of bacteriocin ABC transporters are cytoplasmic^47^, and this domain can also function in isolation from the transporter^48^, so either gene could potentially provide machinery for leader cleavage of the Spb peptides. If operational, this ‘release by lysis’ mechanism would be functionally analogous to colicin release by *E. coli*^49^. Ongoing experiments are focused on elucidating this potential process, and which specific gene(s) possess immunity function.

The *sil* locus was first characterized from *S. pyogenes* strain JS95 (serotype M14) using a modified signature-tagged mutagenesis (STM) approach termed polymorphic-tag-length-transposon-mutagenesis (PTTM). A mutant was identified with a transposon insertion within the *silC* gene, and along with additional specific mutations within the *sil* locus, this work demonstrated that strains with mutations within the *sil* locus were severely attenuated for invasive and fatal mouse infections^23^. Kizy and Neely^50^ also utilized an STM approach with the M14 serotype *S. pyogenes* HSC5 in a zebrafish necrotizing fasciitis model. This study identified attenuated strains with transposons in both the *silB* and *silC* genes^50^, encoding the histidine kinase of the *sil* two-component system and the signaling peptide, respectively^22^. Including our work presented here, there are now three independent *in vivo* genomic selection systems that have each identified genes linked to the *sil-spb* locus in *S. pyogenes*. The *sil* locus, and the *spb* bacteriocin genes, have also recently been shown to be inducible through sensing of host cell asparagine production using the TrxSR two-component system, via a streptolysin dependent mechanism; however, regulation of the bacteriocin genes required a functional *sil* locus^51^. We thus favor that, when present, a functional *sil* locus contributes in a significant way to the invasive character of *S. pyogenes* as previously shown^23^, but that this locus may also add an additional regulatory circuit based on quorum sensing to the *spb* operon to compete with endogenous bacteria in the context of non-invasive infections. However, we also suggest that additional regulatory circuits that respond to the *in vivo* nasopharyngeal environment also control the *spb* operon independently of *sil*, and likely other important functions, to allow for the success of *S. pyogenes* as a globally prominent pathogen.

## Methods

### Bacterial strains and growth conditions

Cloning was carried out in *Escherichia coli* XL1-Blue (Stratagene) grown on BHI plates supplemented with 1.5% agar, or LB broth. Media was supplemented with erythromycin (150 μg/mL) or ampicillin (100 μg/mL) when required. Recombinant proteins were expressed from *E. coli* BL21(DE3) at room temperature. *S. pyogenes* MGAS8232^29^ was grown statically at 37 °C in Todd-Hewitt Yeast (THY) broth, or on solid THY media containing 1.5% agar supplemented with erythromycin (1 μg/mL) and/or tetracycline (0.5 μg/mL), as appropriate. *Lactococcus lactis* MG1363^52^ was grown statically at 30 °C in M17 medium containing 1% glucose (GM17) and erythromycin was used at 5 μg/mL.

### General DNA manipulations

Plasmids and primers used in this work are listed in Tables 2 and 3, respectively. Plasmid DNA was isolated using the Qiagen Miniprep Kit following the manufacturer’s instructions. Digestions were carried out utilizing restriction enzymes provided by New England Biolabs or Roche following the manufacturer’s instructions. Ligations were performed using T4 DNA Ligase (New England Biolabs). *E. coli* cells were made competent using the RbCl_2_ method^53^.

For transformation of *S. pyogenes* MGAS8232, an overnight culture was inoculated 1:50 in THY containing 0.6% glycine. Cells were grown for 2 hours and hyaluronidase was added (1 mg/mL). Cells were grown to OD_600_ of 0.25–0.3 (~3 h). Bacteria were washed once in 15% glycerol, and concentrated 50× in 15% glycerol. Aliquots (200 μL) were stored at −80 °C. DNA (2 μg) was added to room temperature thawed cells in 2 mm electroporation cuvettes, and pulsed with 2100 V and a pulse length of 1.1 ms using the square wave setting (GenePulser, BioRad). Bacteria were transferred to 10 mL THY and recovered at 37 °C. After 2 hours, 1/50 concentration antibiotic was added, and 4 hours later the bacteria were plated on THY agar containing the appropriate antibiotic and grown overnight at 37 °C.

To extract total DNA from *S. pyogenes,* 2 mL of overnight culture was washed twice with 0.2 mM sodium acetate. The pellet was resuspended in 500 μL Tris EDTA Glucose buffer (10 mM Tris, 2 mM EDTA, 25% glucose) containing 1 mg/mL lysozyme and 50 U mutanolysin and incubated for 1 hour at 37 °C. Cells were pelleted and resuspended in 500 μL lysis buffer (50 mM EDTA, 0.2% SDS) with 40 μg proteinase K and 100 μg RNase, and incubated at 65 °C for 2 hours. Next, 50 μL of 5 M potassium acetate was added to precipitate proteins. The supernatant was mixed with 500 μL ice cold 95% ethanol to precipitate DNA. After one wash with 70% ethanol, DNA was dried and resuspended in 100 μL Qiagen Elution Buffer (EB).

### Construction of the *loxP*-*tetR*-*loxP* cassette and integration in the *S. pyogenes* genome

To identify a neutral site within the MGAS8232 genome for integration of exogenous DNA, we utilized a previous bioinformatic analysis of Rho-independent terminators in *Firmicutes*^16^ to locate two opposing genes with separate Rho-independent terminators (*pepO* and *tsf*). This location was used to design two regions of ~500-bp flanking this region for homologous recombination of the RIVET cassette into the genome. The downstream recombination site was PCR amplified from the MGAS8232 genome and ligated into the XhoI and ClaI sites of pG^+^host5. Next, the upstream recombination amplicon was inserted into the XbaI and SacII sites. In order to incorporate the *loxP* sites, these sequences were included into the ‘outside’ primers. In addition, in an attempt to generate a counter selection system, the human herpes simplex virus-1 *thymidine kinase (HSV-tk*) gene was codon optimized for *S. pyogenes* and synthesized by Genscript. This was first cloned into pTRKL2 to fuse the MGAS8232 Gyrase A promoter (P_*gyrA*_) to the *HSV-tk* gene. P_*gyrA*_ was amplified from MGAS8232 DNA and cloned using BamHI and NcoI while *HSV-tk* was amplified from pUC57::*HSV-tk* (Genscript) and cloned using NcoI and XbaI. P_*gyrA*_::*HSV-tk* was cloned into pG^+^host5 utilizing BamHI and XbaI. Lastly, *tetR* was amplified from pDG1515 and cloned using BamHI and EcoRV to create the final cassette. Clones were verified with sequencing at each stage and for the final construct. This final construct was designated pCAS*tet*4.

Following electroporation of pCAS*tet*4 into MGAS8232, cells were grown at 30 °C in liquid media for 4 days, replacing media every 24 hours. Next, cells were shifted to 40 °C to prevent plasmid replication, and grown with erythromycin to select for cells that had integrated the plasmid into the chromosome. Cells were grown for an additional 4 days under erythromycin selection changing media every 24 hours, and plated for single colonies. These clones were grown individually, genomic DNA extracted, and PCR was used to ensure integration of the pCAS*tet*4 construct into the chromosome. Once confirmed, clones were grown in liquid culture at 30 °C for 4 days without erythromycin, replacing media every 24 hours. The culture was again plated for single colonies and patched onto plates with and without antibiotics to isolate colonies that have lost the plasmid. Individual clones were then screened by PCR and the correct integration was designated *S. pyogenes* Cas2.

### Generation of the *S. pyogenes* promoter library and removal of *in vitro* activated promoters

In order to generate a promoter library driving expression of Cre, we first PCR amplified the *cre* gene from pSHE11 and cloned the amplicon into the low-copy Gram-positive/*E. coli* shuttle vector pTRKL2. Next, total genomic DNA from MGAS8232 was digested with increasing concentrations of Sau3AI (0, 0.25, 0.5, 1, 2, 4 Units of enzyme) for one hour. Digestions were then ligated into the BglII site of pTRKL2::*cre*. Ligations were transformed into *E. coli*, and all colonies were scraped off plates, concentrated, and plasmids isolated *in toto*. Plasmids were then transformed into *S. pyogenes* Cas2. Cells were grown in liquid medium overnight in the presence of erythromycin and tetracycline for 24 hours to remove *in vitro* active promoters. The CFU was determined and cells were frozen at −80 °C for further *in vivo* experiments.

### Identification of *in vivo* induced promoters in *S. pyogenes*

Animal experiments were performed in accordance with guidelines established by the Canadian Council on Animal Care and approved by the Animal Use Subcommittee at Western University (Protocol #2009–038). Using 10^8^ cells from batches in which *in vitro* promoters were removed, cells were warmed to room temperature for 30 minutes and inoculated through the nasal route into C57BL/6 mice that express both human HLA-DR4 and HLA-DQ8 mice, as described^54^. After 48 hours, mice were sacrificed and the complete nasal passages were removed, homogenized and plated. Colonies were enumerated on THY agar containing erythromycin, and patched onto THY agar plates containing tetracycline to screen for the loss of the cassette. Tetracycline sensitive clones were subsequently grown, DNA was extracted and transformed into *E. coli* to purify plasmids, and inserts sequenced. Homology searches of potential promoter regions were performed using the Basic Local Alignment Search Tool (BLASTn) tool from the National Center for Biotechnology Information (http://www-ncbi-nlm-nih-gov).

### Quantitative real-time PCR (qRT-PCR)

qRT-PCR analysis of the RIVET cassette integration site, and *spbM* transcripts from both *in vitro* and *in vivo* grown MGAS8232, were determined. For the RIVET cassette, wild-type MGAS8232 and MGAS8232 Cas2 were grown to post-exponential phase. For *in vitro* grown cells, MGAS8232 was grown in THY broth to an OD_600_ or ~0.2 or ~0.9 representing early and late exponential phase, respectively. For *in vivo* grown cells, mice were infected nasally with ~10^8^ CFUs of wild-type MGAS8232 as described above, and the complete nasal turbinates were harvested at 48 h, as described^54^. Cells from these conditions were resuspended in RNAprotect reagent (Qiagen) and total RNA was prepared using the RNeasy Mini Kit (Qiagen). cDNA was synthesized using SuperScript II reverse transcriptase and random primers (Invitrogen). PCR reactions were conducted using iQ SYBR Green Supermix (Bio-Rad), and performed with the Rotor-Gene Real-Time Analyzer (Corbett Life Science) with primers listed in Table 3. Transcripts were normalized against the expression of the *proS* or *gyrA* housekeeping genes^55^.

### Cloning, expression and testing of recombinant Spb peptides

The wild-type *spbM* and *spbN* mature coding sequences (described below) were PCR amplified from MGAS8232 chromosomal DNA and individually cloned into the pET32a expression vector (Novagen). The forward primers amplified each *spb* gene lacking the sequences that encoded the predicted double-glycine type leader peptides. The forward primers were also designed to encode an N-terminal His_6_ tag, followed by a recognition sequence for the tobacco etch virus (TEV) protease cleavage site (ENLYFQ↓G). The resulting plasmids (pET32a::*spbM* and pET32a::*spbN*) generated N-terminal translational fusions with the pET32a thioredoxin tag, a His_6_ tag, as well as the TEV site for removal of the purification tags. SpbM and SpbN were expressed from *E. coli* BL21(DE3) at 25 °C following induction with 100 μM IPTG at 30 °C. Recombinant proteins were solubilized in 8 M urea, and refolded using step gradients into 20 mM Tris-HCl, pH 7.4, 200 mM NaCl. N-terminal tags were cleaved with autoinactivation-resistant His_7_::TEV^56^. To test for antimicrobial activity, SpbM and SpbN peptides (~1 μg) were spotted into wells punched into agar plates inoculated with indicator strains. The two-peptide nature of the bacteriocin was tested by placing the two peptides at increasing distances on the agar plate to observe for functional complementation.

### Cloning and expression of Spb immunity function in *Lactococcus lactis*

A 489-bp amplicon located immediately downstream of *spbN* was PCR amplifed from MGAS8232 and cloned into the XmaI and SphI restriction sites of the lactococcal expression plasmid pMG36e to create pMG36e::*spbI*/RS02360. Plasmids were electroporated into *L. lactis* MG1363 as described^57^. In order to test immunity function, recombinant SpbM and SpbN peptides were spotted into a well punched into agar plates inoculated with *L. lactis* MG1363 containing either pMG36e or pMG36e::*spbI*/RS02360 and zones of inhibition were visualized.

## Additional Information

**How to cite this article**: Armstrong, B. D. *et al*. Identification of a two-component Class IIb bacteriocin in *Streptococcus pyogenes* by recombinase-based *in vivo* expression technology. *Sci. Rep.* **6**, 36233; doi: 10.1038/srep36233 (2016).

**Publisher’s note:** Springer Nature remains neutral with regard to jurisdictional claims in published maps and institutional affiliations.

## Supplementary Material

Supplementary Information

## Figures and Tables

**Table 1:** Potential ‘typical’ sense promoters and corresponding genes identified from the *in vivo S. pyogenes* RIVET screen.

Clone	Corresponding Gene^a^	Description
IVI49	*SPYM18_RS09645*	chromosome segregation protein
IVI53	*SPYM18_RS05290*	amino acid ABC transporter, periplasmic amino-acid binding protein
IVI60	*SPYM18_RS09540*	hypothetical protein
IVI72	*SPYM18_RS03375*	tagatose-6-phosphate aldose/ketose isomerase
IVI84	*SPYM18_RS08645*	conserved hypothetical protein
IVI87	*SPYM18_RS08245*	pyruvate formate-lyase
IVI100	*SPYM18_RS03315*	hypothetical protein, phage associated
IVI156	*SPYM18_RS02350*	putative BlpM homologue
IVI176	*SPYM18_RS01890*	conserved hypothetical protein

^a^Gene nomenclature refers to the *S. pyogenes* MGAS8232 annotation (NCBI Reference Sequence: NC003485.1)^29^.

Further details are provided in Table S1.

**Table 2:** Plasmids.

Plasmid	Relevant description	Source/Reference
pDG1515	source of *tetR*	58
pSHE11	source of *cre*	Paul Sadowsky
pG^+^host5	Temperature sensitive Gram-positive bacteria-*E. coli* shuttle vector, Erm^R^	17
pCAS*tet*4	pG^+^host5 containing the *loxP-tet*^*R*^*-loxP* cassette inserted into the genome of *S. pyogenes* MGAS8232, Erm^R^	This study
pMSP3535	Shuttle vector with nisin inducible promoter (P_*nis*_), Erm^R^	18
pMSP3535::*cre*-forward	pMSP3535 containing *cre* cloned in forward orientation relative to the P_*nis*_ promoter	This study
pMSP3535::*cre-*reverse	pMSP3535 containing *cre* cloned in reverse orientation relative to the P_*nis*_ promoter	This study
pTRKL2	Low-copy Gram-positive/*E. coli* shuttle vector, Erm^R^	19
pTRKL2::*cre*	pTRKL2 containing promoterless *cre*	This study
pTRKL2::P_*spbM*_::*cre*	pTRKL2::*cre* containing a 984-bp *spbM* promoter fragment	This study
pET32a	Protein expression vector containing an N-terminal thioredoxin tag, Amp^R^	Novagen
pET32a::TEV::*spbM*	pET32a containing coding sequence for mature *spbM*	This study
pET32a::TEV::*spbN*	pET32a containing coding sequence for mature *spbN*	This study
pMG36e	*Lactococcus lactis* Expression vector, Erm^R^	31
pMG36e::*spbI*/RS02360	pMG36e containing the 2 ORFs immediately downstream of *spbN* and driven by the P32 promoter	This study

**Table 3:** Primers.

Primer	Primer Sequences (5′ to 3′)^a^^,^^b^
Primers for generation of the RIVET cassette	
* tsf* for XhoI	CGCCTCGAGACTTGCTCAATTGAACCACG
* tsf* rev *loxP* ClaI	GCGATCGAT**ATAACTTCGTATAGCATACATTATACGAAGTTAT**CCGTTTTGACACAACAAAAAGA
* tet* for BamHI	CGCGGATCCAGATAAAAAGTTGATCTTTGTGAAAAC
* tet* rev HindIII	CGCAAGCTTTTAGAAATCCCTTTGAGAATGTTT
* *PgyrA for BamHI	CGCGGATCCGCAAAAGCTCATACGGTCTT
* *PgyrA rev HindIII, NcoI	GCGAAGCTTCCATGG GATCTTGCATTTAAGGAATGCTC
* tk* for NcoI	GCGCCATGGCTTCATACCCATGTCA
* tk* rev XbaI	CCCTCTAGATTAGTTAGCTTCACCCATTTCACG
* pepO* for *loxP* XbaI	GCGTCTAGA**ATAACTTCGTATAATGTATGCTATACGAAGTTAT**ACACCAATAAGGAAGCAAAAA
* pepO* rev SacII	GCGCCGCGGAGCCTAAATGATTGGTGGA
Primers for generating the cre containing plasmids	
* cre* F for pMSP XbaI	GCGCTCTAGAAAGGAGGCACTCAAAATCTCCAATTTACTGACCGTA
* cre* R for pMSP PstI	GCGCCTGCAGCTAATCGCCATCTTCCAGCAG
* cre* F rev pMSP XbaI	GCGCTCTAGAAAGGAGGCACTCAAAATCTCCAATTTACTGACCGTA
* cre* R rev pMSP PstI	GCGCCTGCAGCTAATCGCCATCTTCCAGCAG
* cre* F pTRK PstI	GCGCCTGCAGAAGGAGGCACTCAAAATGTCCAATTTACTGACCGTA
* cre* R pTRK SmaI	GCGCCCCGGGCTAATCGCCATCTTCCAGCAG
Primers for generating the SpbM and SpbN expression plasmids	
* *SpbM-Forward-KpnI-pET32a	CTGGGTACCGGTGGTGGCTCCGGTGAAAACTTGTATTTCCAAGGCAAGAATAATTGGCAGGCAAATGTCA
* *SpbM-Reverse-BamHI-pET32a	GCGGGATCCCTAGAAGCCGCCTGTAGCTCCT
* *SpbN-Forward-KpnI-pET32a	CTGGGTACCGGTGGTGGCTCCGGTGAAAACTTGTATTTCCAAGGCAACGCTCCAGGAGATGCTGTAA
* *SpbN-Reverse-BamHI-pET32a	GCGGGATCCTTAATTTGCTGTATATCCCAAATATGC
Primers for cloning the Spb immunity function	
* spbI*_For_XmaI	GCGCCCCGGGTGGAGGTTGGTATATATGATCTATACGATTTTTATTATTATGAATGC
* *RS02360_Rev_SphI	GCGCGCATGCTTAGTGAGGCCAGATAATATTTGTAGC
Primers for qRT-PCR analysis	
* pepO* RT for	ATTCTGAGCCTTCCTCACGA
* pepO* RT rev	CGAAGAAGGCAACGAAAAAG
* tk* RT for	GCTCCACCACCAGCTCTTAC
* tk* RT rev	GGTCGATGTGACGGTCTTCT
* tsf* RT for	GGCGTTATGGACGCTAAAAA
* tsf* RT rev	TGCGTTTACCAATTCAACGA
* proS* RT for	GGCCATTACACCAGCTCG
* proS* RT rev	TCTCAAGGTTGGCAGCGT
* spbM* RT for	CCAAGGTTGAGGGTGGTAAG
* spbM* RT rev	CCGCCTGTAGCTCCTGTTAC
* gyrA* RT for	ATACTGAAGCGCGCATGA
* gyrA* RT rev	AGGCGGAATGTTAGTTGC

^a^restriction sites or nucleotides encoding the TEV cleavage site are indicated in the primer name and underlined in the primer sequence.

^b^loxP sites shown in bold.
